# Not so FAST. Commentary on the article “Appraisal of the faecal haemoglobin, age and sex test (FAST) score in assessment of patients with lower bowel symptoms: an observational study”

**DOI:** 10.1186/s12876-020-01322-y

**Published:** 2020-07-20

**Authors:** Joaquín Cubiella

**Affiliations:** Department of Gastroenterology, Complexo Hospitalario Universitario de Ourense, Instituto de Investigación Sanitaria Galicia Sur, Centro de Investigación Biomédica en Red Enfermedades Hepáticas y Digestivas, C/ Rúa Ramón Puga 52-56, 32003 Ourense, Spain

**Keywords:** Colorectal cancer, Faecal haemoglobin, Colonoscopy, Prediction models

## Abstract

A recent study by Digby et al. in BMC Gastroenterology evaluated the faecal haemoglobin, age and sex test (FAST) score in the assessment of patients with lower bowel symptoms attended in primary healthcare. This article is a valuable source of information but the conclusions must be carefully assessed. Authors evaluated the FAST score threshold with a 99% sensitivity (≥ 2.12) for colorectal cancer (CRC). Although the number of patients meeting this criteria is high, 53.5% of the patients not referred initially to secondary healthcare, the results on the patients referred to colonoscopy validate the prediction model. The sensitivity and the specificity for CRC detection were 99.0 and 23.2% with a positive and negative predictive value of 8.0 and 99.7%. Additionally, the sensitivity and the specificity for significant bowel disease were 96.1 and 26.2% with a positive and negative predictive value of 24.3 and 96.1%, consistent with our initial results. To conclude, although we need the information regarding the risk of CRC in those patients not referred to colonoscopy, a FAST Score < 2.12 allows to determine a group of patients with a low risk of CRC detection that requires no further evaluation.

## Main text

Colorectal cancer (CRC) diagnosis in symptomatic patients attended in primary healthcare is like looking for a needle in a haystack: symptoms are extremely frequent but CRC is rare [1]. The diagnosis of CRC is based on colonoscopy, an invasive technique with limited availability and associated with severe complications in 1/1000 patients [2]. The delay in the diagnosis may worsen prognosis [3] or, at least, produce anxiety. Two practical questions need to be answered: 1.Is the risk of CRC detection so high that patient should be evaluated in a short period of time, i.e. two week time? 2. Is the risk so low that we can assure no additional evaluation is required? With respect to the first question, health systems have proposed several alternatives, mainly based on symptoms, with limitations on its performance. In this sense, the National Institute for Health and Care Excellence (NICE) referral guideline for suspected cancer NG12 proposes to refer to further evaluation those patients with at least a 3% risk of CRC [4]. In this respect, faecal immunochemical test (FIT) has proven a realistic and objective option to evaluate symptomatic patients and to determine those patients with higher risk [5]. But, on the other side, no strategy has been proposed to exclude further evaluation mainly due to the limited discriminatory ability of the available strategies, including FIT. In this sense, we proposed that symptomatic patients should not be referred to colonoscopy if the risk of CRC detection is similar to the risk of severe complications associated to colonoscopy [6, 7].

In the last years, we have developed and, afterwards, validated two CRC prediction models in symptomatic patients referred to colonoscopy based on demographics, symptoms, blood determinations and FIT [6, 7]. COLONPREDICT is a complex prediction model based on eleven variables -age, sex, faecal and blood haemoglobin (Hb), carcinoembryonic antigen, acetylsalicylic acid treatment, previous colonoscopy, rectal mass, benign anorectal lesion, rectal bleeding and change in bowel habit with an area under the curve (AUC) of 0.92 in the derivation and validation cohorts [6]. In contrast, FAST Score, developed in collaboration with the Dundee team, is an easy to calculate prediction model based solely on the faecal Hb concentration, age and sex. The AUC obtained in the same cohorts where COLONPREDICT was developed and validated was 0.88 [7]. Both prediction models were designed with two thresholds based on 90 and 99% sensitivity. The first threshold (COLONPREDICT ≥5.60, FAST ≥4.50) defines a high risk group in which 90% of CRCs are detected. On the contrary, the second threshold (COLONPREDICT< 3.50, FAST < 2.12) defines a low risk group in which 1% of CRC would be detected and, on account of a negative predictive value higher than 99%, no further evaluation should be recommended. The results in the validation cohort were promising and, as an example, 42.4% of the patients referred to colonoscopy met the low risk criteria according to COLONPREDICT Score, had a negligible risk of CRC and could be evaluated without performing a colonoscopy. In contrast, in the validation cohort of the FAST Score, only 18.8% were classified as low risk. Although these prediction models provide an opportunity for an objective evaluation of the risk of CRC detection and the allocation in the proper diagnostic strategy, our results must be validated in prospective primary healthcare studies evaluating symptomatic patients.

The study conducted by Jayne Digby and colleagues [8] is aimed at evaluating the diagnostic performance of FIT in symptomatic patients evaluated in primary healthcare. The authors present data from the first year of routine use of f-Hb in NHS Tayside, Scotland in the evaluation of patients with new bowel symptoms [9]. Within this study, the authors have performed the validation of the FAST Score in primary healthcare. The authors show the findings related to the number of patients that met a FAST score > 2.12 and the diagnostic performance in those patients referred to colonoscopy. The good news is that this analysis validates our results. In the 1569 referred to colonoscopy (either immediately or at a later date), 1227 (78.2%) had a FAST ≥2.12. The sensitivity and the specificity were, as expected, 99.0% (98/99) and 23.2% (341/1470) with a positive predictive value of 8.0% and, more importantly, a negative predictive value of 99.7%. So, patients with a FAST score < 2.12 had a 0.3% risk of CRC detection, within the expected ranges that allow to exclude referral to colonoscopy. The bad news is that, as authors clearly highlight, 53.5% of the patients not referred initially to secondary healthcare, had a FAST Score ≥ 2.12. In this sense, we miss the information regarding the follow-up of the 3803 patients not referred to colonoscopy during a 2–3 year period in order to identify those undetected CRC. This information would allow to validate, or at least recalibrate, the FAST Score in this population. But we must keep in mind that the threshold evaluated was designed to identify, at least 99% of CRC and to select a group of patients that do not require further evaluation. In this sense, with the available results, we can confirm that patients with a FAST score < 2.12 have a CRC risk very similar to the risk of colonoscopy complications and, thus, colonoscopy should not be recommended.

I would like to add several comments to the results of this article. First of all, I miss the information regarding the diagnostic accuracy of the threshold with a 90% sensitivity (FAST ≥4.50). As previously commented, this threshold was designed to define a high risk group that should be referred immediately for further evaluation. It would be very helpful to know how many patients met this criteria and the sensitivity and positive predictive values in contrast with the f-Hb ≥ 10μgr/gr of faeces recommended in the NICE guideline [10]. It would also be of help to know the positive predictive value for CRC of the patients in the intermediate risk group (FAST Score between 4.50 and 2.12) as long these patients probably benefit from a clinical reevaluation (wait and see strategy) as the authors highlight.

Authors have evaluated the diagnostic accuracy of the FAST Score to detect significant bowel disease (SBD). In the original articles, we validated the first prediction models for the detection of SBD. The results presented by Digby et al. are consistent with our initial findings. In the patients referred to colonoscopy, the sensitivity and the specificity were 96.1% (299/311) and 26.2% (928/1258) with a positive predictive value of 24.3% and, a negative predictive value of 96.1%. I would highlight the difficulty of the non-invasive diagnosis of SBD. Besides, the prognosis or the response to therapy in patients with SBD (advanced adenomas, serrated lesions or inflammatory bowel disease) is not clearly affected by the delay in the diagnosis.

To conclude, the analysis published by Digby and colleagues [8] validates the diagnostic performance of the FAST Score in symptomatic patients referred to colonoscopy for CRC detection. However, its applicability is limited due to the high positivity rate at the 2.12 threshold and the limited sensitivity for SBD detection. Additional information regarding follow-up in those patients without colonoscopy is required in order to confirm the FAST Score < 2.12 as a criteria to avoid unnecessary colonoscopies.

## Data Availability

Not applicable.

## Abbreviations

AUC: Area under the curve
CRC: Colorectal cancer
FAST: Faecal haemoglobin, age and sex test
NICE: National Institute for Health and Care Excellence
FIT: Faecal immunochemical test
SBD: Significant bowel disease

## References

[1] Vega P, Valentín F, Cubiella J (2015). Colorectal cancer diagnosis: pitfalls and opportunities. World J Gastrointest Oncol.

[2] Cubiella J, Marzo-Castillejo M, Mascort-Roca JJ, Amador-Romero FJ, Bellas-Beceiro B, Clofent-Vilaplana J (2018). Clinical practice guideline. Diagnosis and prevention of colorectal cancer. 2018 update. Gastroenterol Hepatol.

[3] Alonso-Abreu I, Alarcón-Fernández O, Gimeno-García AZ, Romero-García R, Carrillo-Palau M, Nicolás-Pérez D (2017). Early colonoscopy improves the outcome of patients with symptomatic colorectal cancer. Dis Colon Rectum.

[4] National Institute for Health and Clinical Excellence. Suspected cancer: recognitioin and referral [Internet]. NICE Guidel. 2017 [cited 18 Mar 2018]. Available from: https://www.nice.org.uk/guidance/ng12.

[5] Pin Vieito N, Zarraquiños S, Cubiella J (2019). High-risk symptoms and quantitative faecal immunochemical test accuracy: systematic review and meta-analysis. World J Gastroenterol.

[6] Cubiella J, Vega P, Salve M, Diaz-Ondina M, Alves MT, Quintero E (2016). Development and external validation of a faecal immunochemical test-based prediction model for colorectal cancer detection in symptomatic patients. BMC Med.

[7] Cubiella J, Digby J, Rodríguez-Alonso L, Vega P, Salve M, Díaz-Ondina M (2017). The fecal hemoglobin concentration, age and sex test score: development and external validation of a simple prediction tool for colorectal cancer detection in symptomatic patients. Int J Cancer.

[8] Digby J, Strachan JA, Mowat C, Steele RJC, Fraser CG (2019). Appraisal of the faecal haemoglobin, age and sex test (FAST) score in assessment of patients with lower bowel symptoms: an observational study. BMC Gastroenterol.

[9] Mowat C, Digby J, Strachan JA, McCann R, Hall C, Heather D (2019). Impact of introducing a faecal immunochemical test (FIT) for haemoglobin into primary care on the outcome of patients with new bowel symptoms: a prospective cohort study. BMJ Open Gastroenterol.

[10] Quantitative faecal immunochemical tests to guide referral for colorectal cancer in primary care [Internet]. [cited 2017 Mar 11]. Available from: https://www.nice.org.uk/guidance/dg30.

